# A systematic review on the impact of leg ulceration on patients' quality of life

**DOI:** 10.1186/1477-7525-5-44

**Published:** 2007-07-25

**Authors:** Oliver R Herber, Wilfried Schnepp, Monika A Rieger

**Affiliations:** 1 Competence Centre for General Medicine and Outpatients' Care & Institute of Nursing Science, University of Witten/Herdecke, Witten, North-Rhine Westphalia, Germany; 2 Chair of Family-oriented and Community-based Nursing, Institute of Nursing Science, University of Witten/Herdecke, Witten, North-Rhine Westphalia, Germany; 3 Research Department, Competence Centre for General Medicine and Outpatients' Care, Faculty of Medicine, University of Witten/Herdecke, Witten, North-Rhine Westphalia, Germany

## Abstract

**Background:**

A systematic review was conducted to analyse journal articles that describe or measure the impact of leg ulceration on patients' quality of life (QoL) in order to improve the content of an educational programme that aims to enhance self-care agency in leg ulcer patients.

**Method:**

Original articles published in English and German between 1990 and 2006 were included if the findings were analysed at the level of patients. Articles were excluded if (1) they investigated the impact of specific treatments or settings on QoL or (2) focused mainly on arterial ulcers or diabetic foot ulcers.

**Results:**

Twenty-four original research articles met the inclusion criteria; 11 studies used a quantitative, 11 studies a qualitative, and 2 used a mixed method approach. The findings were collapsed into 5 core domains. Quantitative studies commonly investigated the parameters of pain, sleep, social isolation, and physical mobility. Patients had significantly more pain, more restrictions regarding social functioning, less vitality, and limitations with respect to emotional roles compared to the respective controls. Other problem areas identified were restrictions in work capacity, recreation, social interaction, psychological well-being, as well as problems caused by treatment regimes. Inconclusive results were obtained regarding pain intensity, physical restrictions, and gender effects.

**Limitations:**

Numerous original studies neither undertook a differentiation of participants by ulcer aetiology nor did they analyse the results according to gender differences.

**Conclusion:**

As leg ulceration has an impact on QoL, national guidelines on the treatment of leg ulceration need to more specifically address these far-ranging effects identified in this review.

## Background

The care of patients with chronic diseases is the focus of many researchers from different academic disciplines. One question they try to answer is how health professionals can improve the life and well-being of chronically ill patients. In a co-operative research project between the medical and nursing profession we developed a nurse-led education programme that aims to enhance self-care agency in leg ulcer patients. One of the primary outcome measures of the intervention in that multi-site clinical trail (ISRCTN42122226) is the assessment of quality of life (QoL) in these patients. In order to improve the content of the educational programme we performed a literature review to describe leg ulcer-related problems among patients with ulcers of venous or mixed aetiology. We conducted a systematic review to analyse articles that describe or measure the impact of leg ulceration on patients' QoL. This was done with the aim of producing a comprehensive overview of the problems so as to provide nursing care directly related to these problems. Although we are aware of earlier reviews, e.g. by Persoon et al. [1] and Wilson [2], there was a need to update this information and to expand it by including German studies. This article encompasses key quantitative and qualitative research focusing on QoL.

## Method

Eligible articles published in journals were identified through the following electronic online databases: MEDLINE via PubMed, and CINAHL (October 1982 et seq.) using the MeSH-term and/or text word search or combinations of: chronic venous insufficiency, leg ulcer*, pain*, restriction, mobility, body image, psycho*, and quality of life [Note that the asterisk (*) is a truncation symbol to search a term in a text to uncover articles with "ulcer", "ulceration", and so on in the article or abstract]. Articles reporting on therapies or presenting results of clinical trials were discarded. The search was limited to original articles published in English and German between 1990 and 2006.

According to the search strategy described by Gilbertson & Aldridge [3] we summarise the results from searching the PubMed database (Search date: 20.10.2006), displaying the number of articles retrieved by each strategy. The MeSH-term search for "leg ulcer" [MeSH: noexp] and "quality of life" [MAJR] found matches in 29 articles. When combining MeSH-term and text word search using the same search terms 242 articles were identified. In order to remove references from the previous search results that included the terms "therapy" and "treatment" we used the Boolean operator "NOT". This search yielded 33 articles. In the final *extreme search *according to Gilbertson & Aldridge [3] we combined the MeSH search, the text word search, and the Boolean search retrieving 21 articles. Using the above-mentioned MeSH-term and/or text word search, 83 articles (including duplicates) were identified by this search strategy. We used a similar strategy to search CINHAL.

Additionally, specific periodicals from the university library were hand-searched including *Journal of Wound *Care, *Journal of Clinical Nursing*, and *Advances in Skin & Wound Care*. Following up reference lists from current original papers identified further literature relevant to the topic. Furthermore, references were sought from staff colleagues and experts from the German Network for Quality Development in Nursing (DNQP). The decision for inclusion/exclusion of articles was made on the basis of the full text articles. Articles were only included if the findings were analysed at the level of patients, not ulcers or wounds. In principle, articles were excluded if (1) they investigated the impact of treatments or settings (e.g. leg ulcer clinics) on QoL or (2) focused mainly on arterial ulcers or diabetic foot ulcers. In the end, after checking all abstracts identified, discarding duplicate articles, and reading the full text versions 24 articles were considered for inclusion.

## Results

Twenty-four original research articles met the inclusion criteria. Of these, 11 studies were quantitative, 11 were qualitative, and 2 used a mixed method approach. The findings were combined and categorised into preset domains which either followed Fallowfield [4] and Phillips et al. [5] or emerged from the data. The presentation of the findings is organised according to the following five core domains: impact of leg ulceration on the (1) physical, (2) occupational, (3) social, (4) psychological domain, and (5) "The impact of leg ulcer treatment". Table 1 provides a summary of the quantitative studies including information about the study design, sample size, age-range of participants, ulcer aetiology, and the measurement tools used. The summary of the qualitative studies encompasses information regarding sample size, methods of data collection and analysis, ulcer aetiology and duration, as well as reported patient problems (Table 2).

**Table 1:** Summary of quantitative studies: study design, sample, duration of current ulcer, aetiology, and instruments (n = 13)

**Authors/Country**	**Study design**	**Sample (distribution)**	**Mean age/Age range (years)**	**Aetiology**	**Instruments**
Chase et al. (2000); USA [17]	Descriptive study	21 patients (8♀/13♂) compared with general US population	Mean age: 72 Range: 39–73	Chronic venous leg ulcers (no indication on how ulcer aetiology was determined)	Short-Form Health Survey (SF-36), 10-item venous leg ulcer knowledge test (multiple choice)
Cullum & Roe (1995); UK [7]	Survey; Interviewer-administered semi-structured interview	88 patients (58♀/30♂) and 60 healthy elderly controls (36♀/24♂)	Mean age: 80 Range: 65–98 Mean age (normal population): 77 Range (normal population): 65–91	No information	Nottingham Health Profile (NHP); Life Satisfaction Index; Hospital Anxiety & Depression Scale; Short-form McGill pain questionnaire; Health Locus of Control Scale
Flett et al. (1994); New Zealand [15]	Survey + comparing two groups; Interviewer-administered questionnaire for leg ulcer patients; Self-administered questionnaire for controls	14 patients (10♀/4♂) and 14 controls (8♀/6♂)	No information	Ulcers not classified according to type, size, or chronicity	6-item disability scale (activity and mobility); 5-point scale (for frequency); Medical Problems Scale (diagnostic medical problems); 9-item measure (psychosomatic symptoms); 3 single item measures (health, pain, worry/concern)
Franks & Moffatt (1998); UK [14]	Cross-sectional study (survey); Interviewer-administered questionnaire	758 patients (486♀/272♂) compared with mean age/sex-matched normal population values	Mean age: 74.6 Mean age (normal population): no information	No information	Nottingham Health Profile (NHP)
Gonçalves et al. (2004) Brazil [19]	Cross-sectional study; Interviewer-administered questionnaires	90 patients (49♀/41♂)	Mean age: 61.4 (all patients) Mean age: 60.5 (venous leg ulcer patients)	Venous:(n = 73) 82% Arterial: (n = 1) 1% Mixed: (n = 3) 3% Others: (n = 13) 14%	0–10 numeric pain intensity rating scale; Short-form McGill Pain questionnaire
Hareendran et al. (2005); UK [24]	Questionnaires + individual semi-structured interviews	38 patients (26♀/12♂)	Mean age: 71.4 Range: 46–91	Proven venous leg ulcers (Duplex ultrasound scan)	(modified) Skindex questionnaire
Hamer et al. (1994); UK [6]	Survey; Interviewer-administered semi-structured interviews	88 patients and 70 healthy elderly controls	Age: 65+ Mean age (normal population): no information	No information	Nottingham Health Profile (NHP), Life Satisfaction Index, Hospital Anxiety & Depression Scale, Short-form McGill pain questionnaire, Health Locus of Control Scale
Hofman et al. (1997); UK/Sweden [10]	Longitudinal study; Semi-structured questionnaire	140 patients (87♀/53♂)	Mean age: 64.7 Range: 22–92	Venous:(n = 94) 67% Arterial: (n = 4) 3% Mixed: (n = 9) 6% Others: (n = 33) 24% (using ABPI)	6-point verbal rating scale for Pain (McGill pain questionnaire)
Hyland et al. (1994); UK [20]	Testing of disease-specific self-report questionnaire	50 patients (36♀/13♂ 1 gender unknown)	Mean age: 77 Range: 45–90;	Different aetiologies of leg ulcer	34-item self-report questionnaire on Quality of Life
Klyscz et al. (1998); Germany [18]	Longitudinal study; Self-administered questionnaire	142 patients (93♀/49♂)	Mean age: 51 Range: 16–76	CVI I: (n = 51) 37.5% CVI II: (n = 44) 32.4% CVI III: (n = 41) 30.1%	Tübinger Questionnaire for measuring Quality of Life in patients with CVI (TLQ-CVI)
Lindholm et al. (1993); Sweden [12]	Survey; Postal questionnaire	125 patients (74♀/51♂) compared to normal population	Mean age: 77 Range(♂): 36–91 Range (♂): 37–93 Mean age (normal population): no information	Venous, arterial and mixed aetiology ulcers	Nottingham Health Profile (NHP) only part 1 (pain, physical mobility, sleep, energy, emotional reactions, social isolation)
Phillips et al. (1994); USA [5]	Survey; Standardised personal interviews	62 patients (37♀/25♂)	Mean age: 62 Range: 33–90	Leg ulcers of varying aetiology, size and depth	Standardised personal interviews covering 4 domains (physical, functional, financial, psychological)
Price & Harding (1996); UK [13]	Survey; Comparing chronic leg ulcer patients with healthy controls	55 patients (37♀/18♂)	Mean age: 70.4	Chronic leg wounds of any kind with a minimum duration of 3 months; Exclusion criteria: diabetes, neurological/cardiac disorder, active vasculitis	Short-Form Health Survey (SF-36)

**Table 2:** Summary of qualitative studies: sample, data collection, and reported patient problems grouped according to domains (n = 13)

**Authors/Country**	**Sample/Data collection/Data analysis**	**Aetiology/Ulcer duration**	**Problems experienced by leg ulcer patients with respect to:**				
			
			**Physical Domain**	**Occupational Domain**	**Social Domain**	**Impact of Treatment**	**Psychological Domain**
Bland (1996); New Zealand [25]	9 patients (4♀/5♂); Phenomenological approach	Chronic open leg ulcers (aetiology not specified); Current duration: 8 months – 6 years	Pain Leakage Smell Foot odour	Difficulties maintaining personal hygiene		Difficulties to dry bandages; Difficulties to incorporate recommendations into everyday life; Receiving conflicting information; Unable to comply with treatment regime; Needing larger size shoes because of bulk bandages	Bandages draw other people's attention to the leg, bandages are seen as unsightly, frustration about having to rest for weeks; Concern about job security; Feelings of guilt when unable to comply with treatment regime; Invasion of privacy through nurses, Concordance diminished on long-term basis
Brown (2005a, b) UK [29, 31]	8 patients; Semi-structured, in-depth interviews using an interview guide; Phenomenological approach	Various leg ulcers	Poor mobility		Social disconnected-ness	Bandages restricted mobility; No understanding of the disease; Symptom relief is more important than complete healing; Close relationship with nurses;	Anxiety over falling; Feelings of loneliness; Feelings of depression
Charles (1995); UK [8]	4 patients (1♀/3♂); Semi-standard interviews with open-ended questions; Phenomenological approach	Chronic venous leg ulcer; Duration: 5 – 35 years	Pain Impaired mobility			Health professionals do not: (1) listen to patients concerns, (2) explain treatment regimes, (3) establish empathy	Hopelessness; Helplessness; Loneliness; Loss of self-worth; Social isolation; Reduced self-esteem
Chase et al. (1997); USA [23]	37 patients; participant observation, field notes, pain logs; 7 patients interviewed using open-ended questions; Phenomenological approach		Pain Pruritus Smell Swelling Impaired mobility	Loss of job; Treatment-imposed limitations on activity	Life accommodation		A never-ending healing process; Open ulcer as a reminder of threat to tissue integrity; Body image changes; Limited social contact; Fear of amputation; Powerlessness; Difficulties in wearing shoes & clothes; Loss of freedom
Douglas (2001); UK [11]	8 patients (6♀/2♂); Formal, unstructured interviews; Grounded theory	Venous leg ulcer; Duration: > 1 year	Pain Leakage Smell Impaired mobility Sleeplessness			Perceived conflicting advice by professionals; Seeking alternative treatment options; No understanding of the disease Little knowledge of or control over treatment; Poor adherence to treatment; Relationship with professionals	Expectation; Acceptance; Disappointment; Low self-esteem, Altered body image; Loss of self-control; Effect on relationships
Ebbeskog & Ekman (2001); Sweden [26]	15 patients (12♀/3♂); Age range: 74–89; Personal interviews in form of a dialogue; Phenomenological-hermeneutic approach	Active venous leg ulcer (verified using ABPI > 0.8); Duration: 4 months – 2.5 years	Pain Leakage Impaired mobility Sleep disturbance Loss of energy	Difficulties maintaining personal hygiene;	Visiting friends had to wait until healing; Avoidance of visiting public bathing-places; Reduced social contacts;	Uncomfortable dressings; Difficulties in finding suitable shoes that fitted the bandaged foot	Altered body image; The wound is a constant reminder of the disease; Feelings of having no control over the body; Powerlessness; Feeling of being trapped; Feelings of depression; Difficulties imagining a life without leg ulcer; Fear of recurrence; Feeling that pain killers are bad for the body; Feeling that something bad might happen to the ulcer; Hopeful towards healing;
Hareendran et al. (2005); UK [24]	38 (26♀/12♂); 6 focus groups using an interview guide with open-ended questions; Individual patient interviews for questionnaire development	Venous leg ulcer; Duration: 4 months – 45 years	Pain Discharge Pruritus Sleeplessness	Difficulty bathing;	Limitation of daily living, holiday, and hobbies; Problems with family function	Disappointment with treatment;	Ulcer affected self-confidence and appearance; Increased dependency
Hopkins (2004) UK [28]	5 patients (1♀/4♂); Unstructured interview supplemented by a diary; Interpretative phenomenological analysis	Venous ulceration; Non-healing ulcers of > 1 years			Social exclusion; wasted days; private becomes public;	Good relationship with nurses;	Coping strategies: acceptance, comparison, thinking differently, hope
Hyde et al. (1999); Australia [16]	12 patients (12♀); In-depth semi-structured and follow-up interviews; Gender-specific collection method	Leg ulcer; Duration: > 3 years	Pain Leakage Smell Sleeplessness				Pain as an indication for infection; Concern about analgesic therapy; Embarrassment; Loss of femininity; Maintaining control over integrity of legs; Wearing non-preferred appeal; Loneliness Coping strategies: determination, stoicism, resilience, hope;
Hyland et al. (1994); UK [20]	22 patients; 6 focus groups Data analysis: no information	Different leg ulcer aetiologies	Pain Restrictions of activities			Receiving conflicting information; Cost of bandages	Feelings of regret, depression, loss of will power; Feelings of helplessness; Feeling unclean; Loss of femininity, Preoccupied by the ulcer, Uncertainty of healing; Patients engage in coping strategies
Klyscz et al. (1996); Germany [21]	55 patients; Unstructured interviews; Content analysis	Various stages of chronic venous insufficiency ; CVI I: (n = 18) CVI II: (n = 22) CVI III: (n = 25)	Pain Heavy legs Leg complaints Impaired mobility		Quitted leisure time activities;	Time consuming consultations; Compression bandaging hampered mobility,	Cosmetic problems (e.g. do not wear skirts or elegant shores); Coping strategies: cycling, swimming,, walking, cold shower, leg elevation,
Krasner (1998a, b); USA [22, 27]	14 patients (7♀/7♂); Semi-structured interviews; Hermeneutic phenomenological approach	Active venous leg ulcer & ulcer pain at initial interview; Current duration: 2 months – 7 years	Pain Swelling	Interference with the job		Patients are labelled non-compliant; Patients have difficulty in operationalising professional advice	Carrying on despite the pain; Feelings of depression
Walshe (1995); UK [9]	13 patients (12♀/1♂); Informal unstructured interviews; Phenomenological approach	Venous leg ulcer; Duration: 4 months – 10 years	Pain Leakage Smell Impaired mobility Sleep disturbance	Difficulties in maintaining personal hygiene	Housebound	Questioned efficacy of dressings; Perceived inconsistency of treatment; Little understanding of leg ulceration; Control of treatment given to professionals	Alteration in self-image; Pessimistic view of healing; 4 coping strategies: comparison, feeling healthy, altered expectation, being positive; Uncertainty & worry Difficulties in getting shoes & clothes

### Impact of leg ulceration on the physical domain

The physical domain encompasses numerous aspects of pain as well as pruritus, swelling, discharge, malodour, and various aspects related to mobility. With regards to pain, including pain intensity, the influence of pain on physical activities, sleep, analgesic therapy, and the coping strategies used to reduce pain are described.

### Pain

Pain was described in both quantitative and qualitative studies as the worst thing about having an ulcer [6-11] despite other important medical problems [5]. Generally leg ulcer patients experienced significantly more pain than the controls [12-14] with an increase of pain intensity in larger ulcers [15,7]. A gender analysis revealed that male patients seemed to have more complaints regarding pain than women [5].

### Pain intensity

Hofman et al. [10] reported that 64% of the sample (n = 60) indicated pain levels between 4 (horrible pain) and 5 (excruciating pain) on a 6-point verbal rating scale. However, Chase et al. [17] described a much lower pain incidence. A mere 10% of patients surveyed experienced "severe" pain, 19% had "moderate" pain, 38% had "mild" to "very mild" pain, while 33% indicated "no pain". Pain intensity was higher in patients with a low Ankle Brachial Pressure Index (ABPI) supporting the notion that ulcers of mainly arterial aetiology are more painful [7]. Equally, patients of chronic venous insufficiency (CVI) stage III suffered greater pain intensity than patients of CVI stage I/II [18]. Male patients generally reported significantly higher pain values than women [12]. This was even confirmed when the pain levels were adjusted for normal-matched values indicating poorer perceived health in men [14].

Likewise an analysis of statistical correlations revealed that men experienced greater pain intensity than women [19]. However, Franks & Moffatt [14] argue that differences in gender may vary depending on the method of data manipulation employed. The most intense pain was reported in patients from the lowest income bracket [19]. Qualitative research discovered that pain intensity varied depending on the time and season [20]. In some patients pain intensity was worse in winter while in other patients pain was the worst in summer [20,21]. Interestingly, patients with leg ulcer duration of more than 2 years experienced significantly less pain and were in better general health than patients with a duration of less than 2 years [13]. During the semi-structured interviews, some patients experienced decreased pain as a sign of wound healing, while others experienced increased pain during an infection or swelling [22].

### Pain influences physical activities and causes sleeping problems

Ulcer pain restricted physical activities such as walking [20] and was frequently associated with leg and ankle oedema [23]. Moderate and severe pain levels caused interference with normal productive activities [17]. Although patients stayed in contact with relatives and friends, some were going out less frequently due to pain and discomfort [7]. Leg ulcer pain often occurred at night [16] and prevented patients from getting a full night's sleep [9] which created a negative state of well-being [11]. Discomfort and pain from the ulcerated leg kept some patients awake [7], while others woke up when the effect of pain killers lessened [24]. Hyland et al. [20] found a correlation between pain and sleep loss or thinking about the ulcer. That is, the more pain patients experienced the more they would think about the ulcer and the more sleep loss they had. Sleeplessness was also a major source of exhaustion and worry [8]. However, sleep disturbance was only marginal in women but above "normative scores" in men [12]. Itching also contributed to patients waking up during the night as was stated during interviews [24].

### Analgesics and non-pharmacological therapy

Although pain was the most common cause of functional limitation, not all interviewed patients were prescribed or used pain killers [24]. Pain was often underestimated by physicians as they did not perceive chronic wounds as life-threatening [5]. In the study by Hofman et al. [10] only half of the venous leg ulcer patients (30/60) with pain levels of 4 (horrible pain) and above (excruciating pain) received morphine-based analgesia and 16 (27%) received no analgesia at all. If pain killers were used, in 70% of cases non-steroidal anti-inflammatory drugs were employed [19]. Some of the interviewed patients were prescribed ineffective analgesia [9] or did not report pain to their caregivers [11]. Qualitative research revealed that some patients felt uncomfortable using medication to relieve the nagging ulcer pain while others did not see any other possibility for managing it [16]. Non-pharmacologic practices for pain management included phytotherapeutic drugs, resting, repositioning of the leg, massage, and dressings [19]. Pain experiences were described during interviews as both acute [22] and chronic in nature [23]. Often pain was attributed to the general aging process of the patients. If pain killers did not relieve the intense pain, patients even considered having their leg amputated [20,25].

### Coping strategies for reducing pain and ulcer prevention

Several coping strategies were described in the literature, predominantly in qualitative studies. One strategy to relieve the discomfort caused by pain was getting out of bed and walking around [24]. Engaging in distracting activities was used to prevent excessive preoccupation with the ulcers [20,26]. Avoiding situations which triggered or exacerbated pain, e.g. standing or walking, was another coping strategy [9]. Putting the leg in different positions or doing massage was described as pain relieving for a while [26]. Leg elevation ameliorated the pain for some patients, whereas in others it remained the same or even exacerbated the pain [10]. Yet leg elevation, compression stockings, and diuretic therapy were most effectively in reducing swelling leading to decreased pain levels [22]. When ulcer healing had occurred patients were often very conscious of preventing further ulcers [20,16]. Protecting the leg was a means to maintain some control over its integrity [16]. However, some avoidance strategies caused other deficits. These included avoiding crowded shopping areas, being afraid of having children on the knees, avoiding cats [20], and limiting mobility [9]. Due to a number of these some patients became virtually housebound [9].

### Pruritus, swelling, discharge & smelling

Leg ulceration was often associated with pruritus, discharge, and swelling of the leg. Swelling of the leg did correlate with discharge and impaired mobility [5]. In a study by Klyscz et al. [18] patients with CVI stage I/II mentioned "swelling of the leg" more frequently; however, pain intensity was less than in patients with CVI stage III. Most of the participants in the study by Krasner [27] mentioned that standing increased the swelling which in turn increased pain. Almost 70% of the sample surveyed by Hareendran et al. [24] reported pruritus beneath the bandages or around the ulcer. Although pruritus was a frequent complaint, some patients interviewed interpreted it as a sign of healing, while others construed it as the first alert of recurrence after the ulcer had healed [23]. Hareendran et al. [24] reported that more than 60% of the sample had exudate that smelt or stained. Malodour and discharge was seen as part of leg ulceration [9] causing dismay [7,11] and horror [11].

According to the researchers, 24% of patients seeking treatment had ulcers that smelt unpleasant [7]. Malodorous leg ulcers had a negative effect on patients' social life [7,23,28,24], led to higher anxiety and depression scores, lower life satisfaction [7], and altered body image [11]. Patients attested to being embarrassed about the smell of the ulcer [25], which contributed to the negligence of the ulcer or entire leg [23]. The problem was aggravated by foot odour when toe to knee bandages had to remain in place for a week preventing patients from having a shower [25]. Since coping mechanisms were often inadequate patients felt embarrassed about ulcer leakage [16] and had difficulties in maintaining dignity and outward appearance [9]. Almost half of the patients were of the opinion that other people noticed their ulcer which resulted in significantly lower life satisfaction-scores for these patients [7]. At times, patients reported not leaving their home when the dressings were soaked with fluid from the wound [25]. Coping strategies to control malodour included the use of eau de cologne or putting another bandage on top of the other; wearing slacks was a strategy to hide the dampness of the bandages [16].

### Mobility

As reasons for restricted mobility Hyland et al. [20] identified that (1) the level of pain prevented physical activities, (2) the need for dressing changes acted as a deterrent for outdoor activities, and (3) avoiding strategies hampered patients in moving freely.

### Mobility restrictions and gender/age differences

Several studies showed that the patients' mobility was adversely affected due to leg ulceration in the majority of cases investigated [5,20,15,9,17,29]. In two quantitative studies mobility restrictions were described as the second worst thing about having an ulcer [6,7]. Franks & Moffatt [14] compared Nottingham Health Profile (NHP)-scores of 758 leg ulcer patients with age/sex-matched normal values. Patient scores were significantly higher than the normal values, indicating poorer health, particularly in terms of mobility. Mobility impairment was significantly aggravated in leg ulcer patients with obesity [5]. Klyscz et al. [18] found that CVI stage III patients felt much more impaired with respect to their functional status than patients with CVI stage I/II. As a result of reduced mobility patients went out less frequently [7] and became more dependent on friends and family members [24]. The results were ambiguous regarding gender effects.

### Impact of leg ulceration on the occupational domain

Typical items related to the occupational domain included restrictions in carrying out paid employment, the ability to cope with household duties, and restrictions experienced by the affected person when engaging in personal hygiene.

### Restrictions in work capacity

Employed ulcer patients stated that their work and leisure capacity was restricted, although general health was considered to be good [17]. Restrictions in work capacity were experienced particularly among younger patients, correlating with time lost from work and job loss [5]. Job security became a real concern in patients who could not avoid time off from work [25]. At times mobility restrictions led to an inability to work with the result that patients relied solely on disability payments [23]. This was particularly dramatic in homeless people as they need to be mobile in order to access food and shelter [23]. Although the majority of patients investigated by Cullum & Roe [7] had retired from employment, in some cases retirement was caused by reduced mobility. Among non-working patients, 42% stated that their ulcer contributed to the decision to stop working [5].

### Restrictions in housework

Activities of daily living such as preparing meals or carrying out housework were impeded for the majority of patients [7]. Moreover, leg ulceration caused problems for looking after the home and home life as compared to the control group. To a lesser degree patients mentioned problems regarding climbing stairs or getting on and off busses [20]. Having to leave household chores to partners or being dependent on them provoked a feeling of guilt in some patients [9]. While women with leg ulcers lost their strength for housework, men were more successful in getting help from the home help services [26].

### Restrictions in personal hygiene

Leg ulceration also affected the ability to effectively engage in personal hygiene [11,26]. The majority of patients reported difficulties in washing and bathing [7,9,17,24]. Among the obstacles that prevented patients from conducting personal hygiene was the fear of getting the ulcer wet or spoilt [7,26]. Others stated that the dressing prevented them from taking footbaths and washing their bodies everyday [9,26]. Furthermore, patients were worried that the healing would be disturbed. Some even missed their hygienic routines and postponed visiting a chiropodist to have their nails cut until wound healing occurred. However, friends and family members contributed with practical advice and arrangements like a portable bathtub which aided the execution of personal hygiene [26].

### Impact of leg ulceration on the social domain

The social domain deals with problems caused by the ulcer that affected people's social life. These include restrictions regarding leisure time activities, performing as carers, and having social contacts with friends and family members.

### Social isolation, loneliness and recreation

Social isolation was a common problem for many leg ulcer patients which was provoked by a combination of circumstances [16]. According to Lindholm et al. [12] women with leg ulceration experienced no impact on social isolation compared to the general population whereas men experienced a negative effect. Possible explanations for social isolation were that patients' thoughts constantly revolved around treatment [9] and that restrictions in work capacity hindered patients from making social contacts [8]. The participants in the qualitative study by Brown [29], however, denied feeling lonely but described feeling socially disconnected. Moreover, patients' lives often revolved around nursing visits, which led to exclusion from social activities [28]. Patients were also hampered in pursuing leisure activities such as swimming, gardening, and walking [27,17,24] or were prevented from holidaying or travelling [20,24]. If they did travel they visited an especially intimate friend. Others avoided going to public bathing places as they felt that they could not show their wound in public [26].

In order to maintain recreation, life accommodations had to be made such as modifying the sewing machine for an elbow control [23]. Although 70% of patients surveyed by Cullum & Roe [7] had been able to maintain their hobbies, 43% had given up some of their hobbies. However, many of the patients interviewed found satisfaction in new leisure activities [27]. Leg ulceration hindered patients to perform as carers which was significantly associated with an increased level of anxiety. Even the contact with friends and family members became narrowed to include only the closest ones [7]. Often the only means of keeping in touch was by telephone [26] so that some patients felt largely housebound [20]. Significant relationships were found in leg ulcer patients between going out of their house less frequently and high anxiety scores [7]. Some mobility restrictions were self-imposed by patients [24] and further contributed to an increase in the level of helplessness [9]. Nevertheless, some patients associated good things with having an ulcer such as getting meals on wheels and seeing the district nurse [7,25,23,29]. This supports the notion that some patients experienced benefit from their disease [7,23].

### Impact of leg ulceration on the psychological domain

The psychological domain includes negative emotional reactions caused by the ulcer that gave patients a feeling of being controlled by their disease. The majority of patients had a pessimistic vision of the future and experienced alterations in their body image.

### Negative emotional reactions

Psychological problems included the lack of social contact, feelings of depression, reduced will power, helplessness, and a sense of uncleanliness [20]. Besides that, feelings of guilt, disappointment, and sadness about having an ulcer were expressed during unstructured interviews [11]. Ongoing frustration led some patients to feel depressed [27]. Other patients mentioned feelings of anxiety, social isolation, anger, and decreased self-confidence [5].

In order to hide feelings of depression interviewees put on a cheerful face when they met friends or visited the clinic but cried when particularly lonely [26]. Feelings of frustration occurred when patients had to rest for weeks, particularly among men as they were likely to be the main income earner of the family [5,25]. Contrary to the results described above, one study indicated that leg ulcer patients felt peaceful, happy, and calm as measured by the mental health sub-scale of the SF-36 [17]. It seemed that the psychological impact was greater among leg ulcer patients compared to patients undergoing major invasive procedures [5]. Inconclusive results were obtained for emotional reactions in women. Lindholm et al. [12] found NHP-scores in leg ulcer women that were similar to those in the general population whereas Franks & Moffatt [14] obtained significantly higher scores as measured by the SF-36 indicating a poorer perceived health. Leg ulcer patients had significantly lower levels of self-esteem and more health-related worries than the control group [15]. As the pain was continuous, many patients felt reminded on the ulceration [9]. Due to this constant awareness of the ulcer [7,23] patients were unable to relax which adversely affected their mood [24]. A minority of leg ulcer patients stated that their sex life had been affected as a consequence of the ulcer [7]. Klyscz et al. [21] described how the experience of CVI patients changed from a primarily cosmetic problem (CVI stage I) to a rather complex disease that influenced all aspects of life in patients with CVI stage III.

### Being controlled by the ulcer

All but one patient were aware of the ulcer, predominantly at night [7]. Contrary to the study by Hyland et al. [20] who described that patients spent on average 1.5 hours per day thinking about the ulcer, Hopkins [28] demonstrated how people contained the ulcer. Other patients described a feeling of being committed to the outcome of ulcer healing, but lacked ownership of the condition [23]. During the long process of leg ulceration patients thought they were in a hopeless situation and thus did not seek alternative treatment solutions [8]. Furthermore, patients who had chronic pain often stated that they were out of control and believed that they could not be helped [9]. Patients felt they had no control or power over the ulcer when the expected result of healing did not occur [8,26]. Feelings of "loosing control" and "role reversal" were expressed among family members [11].

### Wound healing and vision of the future

The majority of patients interviewed were pessimistic about ulcer healing [9,11]. Patients were often resigned when wound healing did not take place within a certain period of time [23]. An infectious ulcer caused additional concern about a set-back in the healing process [16]. Conversely, in a sample where more than half of the patients had experienced healing of a previous ulcer, only 3% of leg ulcer patients mentioned worry about healing [6]. However, patients with more than one ulcer were less optimistic about healing [5]. Patients experienced the typical cycle of ulceration as inevitable, as it was mainly attributed to being part of the aging process or family history [11]. Although the expectation of healing reduced with increasing age [9] patients never stopped hoping that one day their ulcers would heal [25]. Patients anticipated healing with a great boost to their morale and improvement in their standard of living [9]. When the ulcer was healed they planned to do all the things they had postponed because of it [26]. However, patients could not contemplate a life without a wound since they had had it for a long time. Others had difficulties to recall what life was like before the ulcer had occurred [28].

### Altered body image

In order to hide the bandages that cover the ulcer some women started to wear trousers in which they felt unattractive [9,16,26,24]. Getting suitable shoes represented another problem as it was difficult to put them on with swollen or bandaged feet and ankles [9,7,25,23,16,26]. The difficulty of finding suitable shoes limited the person's chance of taking the daily walks they were used to [26]. In female patients, having to wear non-preferred clothes or shoes led to a perceived loss of femininity [20,16] and interfered with the women's social life [9]. However, when self-control was regained, body image and self-esteem improved [11]. In the quantitative study by Hyland et al. [20] 32% of patients were of the opinion that their feet dominated their body. Especially among women, frustration and loss of self-identity was noticeable since they felt unable to act according to their traditional role [11]. Among older people, coping with and accepting the situation was more effective because they adapted to restrictions that occur with aging without the loss of self-esteem [5]. Ebbeskog & Ekman [26] reported that patients had accepted themselves as a person with a leg ulcer.

### Impact of leg ulcer treatment

Leg ulcer treatment was experienced as burdensome and time consuming and patients often relied on help. The treatment regime was experienced as uncomfortable and patients felt dissatisfied with the care provided. In addition, patient participation, knowledge deficits, and patient's information seeking behaviour are also discussed in this domain. Lastly, leg ulcer treatment had an adverse effect on patients' financial situation.

### Therapy causing discomfort

Wearing dressings [7] and bandages [25,26] was felt uncomfortable. Therefore some patients took off the bandages to reduce discomfort [26] despite acknowledging that this would interfere with healing [29]. Some of the interviewed patients had a feeling of being trapped by their bandages [26] or felt like a prisoner in their own home [29]. Compression bandages restricted the execution of day-to-day activities [24] and caused other people to draw attention to the leg [25]. The impairment caused by compression hosiery was greater in patients with CVI stage III than compared to patients with CVI stage I/II [18]. Twenty-three per cent of patients mentioned that they occasionally removed stockings and bandages when they were too loose, too hot, or too tight [7]. Qualitative research revealed that leg ulcer treatment such as cleansing and changing the dressing caused pain [9,22,16,11]. In the quantitative study by Hofman et al. [10], however, some patients experienced dressing changes as pain relieving, while others stated the converse. Treating the ulcer was experienced as time consuming [21].

### Satisfaction and problems with care provision

The majority of patients interviewed by Hareendran et al. [24] felt satisfied with the nursing they received. However, some patients were dissatisfied with treatment practices and symptom reduction [24] while others questioned the efficacy of dressings and alterations in treatment regimes [9]. Patients were of the opinion that the suggested treatment regime caused intolerable side-effects [8]. Although some of the patients' needs remained unmet [24] they still tended to leave control in the hands of professionals [9]. Patients complained, however, that professionals did not listen, they did not explain what they were doing, nor did they establish a sense of empathy with the patient's situation [8]. Furthermore, an invasion of privacy through district nurses when they came for their regular visits in people's homes was reported during interviews [25]. Inconsistencies of care or the lack of a regular nurse were further points of critique mentioned [28].

### Patient participation

Patients were likely to accept comprehensive treatment regimes on a short-term basis when healing was within reach. However, compliance diminished on a long-term basis as they gradually came to realise that this was unlikely [25]. Some patients rarely participated in the physical care of the ulcer as they thought they would interfere with the job of the nurse [9]. Hyland et al. [20] discovered that patients did not engage in any more self-care behaviour even if their ulcer deteriorated. Sometimes patients were labelled non-compliant when in fact they were unable to incorporate the professionals' advice into their everyday lives [25,22]. At times patients did not comply with treatment regimes as they felt that the current treatment was not in their best interest [25]. Other patients felt guilty when they were unable to comply with treatment regimes although they had understood the importance of the suggested regimes [25].

### Knowledge deficit and information seeking behaviour

The majority of patients and carers interviewed felt that they had very little knowledge of or control over their treatment with little understanding of the underlying cause of the ulcer [9,11]. Many attributed leg ulceration to a trauma [9,29] or to an underlying condition, while others did not know or could not remember the cause of the ulcer [6,7]. Patients often had a vague idea that "bad circulation" contributed to the persistence of the ulcer but they did not understand the effect of circulation on healing. Leg ulcer wounds were often compared with other wounds and patients wondered why they healed so slowly [9]. Chase et al. [17] found knowledge deficits of leg ulcer patients regarding the cause of venous ulcers, optimal resting position, the benefit of walking, dietary influence on healing, and decision making. However, when patients were asked if they would like to have more information about the ulcer over 50% stated that they did not want any further information [6,7]. Patients and carers often stated that they had received conflicting information from different health professionals [20,25] which caused several participants to seek alternative treatment and caused difficulties around patient compliance [11].

## Discussion

The discussion begins with a comprehensive analysis of the weaknesses and strengths of the review followed by a comparison of our review with two other reviews on the same topic. Subsequently, we shall provide a critical reflection of the studies included in this review. Lastly, we shall elucidate the implications of the findings with respect to clinical nursing practice.

### Weaknesses and strengths of this review

One of the shortcomings of the review was that articles were included in which the authors broadly reported about "leg ulcer patients" but did not specify these according to the ulcer aetiology, depending on which symptoms differ. Other studies, however, reported to have included "venous leg ulcer patients" but did not verify them by means of physical examination. One study talked about the three stages of CVI of which only stage IIIb is an open venous leg ulcer. Although we generally excluded studies that focused on arterial leg ulcers, some studies pooled the results for venous, arterial, and mixed aetiology ulcers in their findings.

The most frequently used measurement tools (e.g. SF-36, NHP) that were employed in the original studies are well validated and frequently used in research [14]. A detailed discussion regarding these measurement tools, however, goes beyond the scope of this article. Nevertheless, for a comprehensive analysis on health-related QoL tools for venous-ulcerated patients we refer the reader to an article by Anand et al. [32]. They provide a good overview of the discriminative and evaluative properties of generic and disease specific instruments commonly used in QoL studies.

The inclusion of baseline QoL results from clinical trials or studies on treatment and therapy was omitted as its reporting in the original studies was often scarce or incomplete. In some cases, baseline data was presented only for such QoL items where the subsequent intervention yielded statistically significant results. In others, post intervention data were expressed in terms of a percentage change compared to baseline data, thus lacking a separate, and, above all, complete description of baseline data. As a result of the incomplete reporting of QoL baseline data in clinical trials, random results could have occurred making the review vulnerable with respect to selection bias.

The wealth of information gathered in this review was accomplished through the combination of quantitative and qualitative articles on the topic. The data analysis from the two methods generated complementary insights that together created a more complete picture of a complex phenomenon. This is in line with Pope & Mays [30] who argued that different methods applied to the same research question are part of the validation process to compare results for convergence. Since the majority of quantitative studies used solely generic measures of health such as the SF-36 or the NHP, many aspects on the impact of leg ulceration would not have been discovered by this approach alone. Generic measures generally reflect a limited perspective of an otherwise very wide and multi-faceted concept such as QoL.

### This review in relation to the review by Wilson [2] and Persoon et al. [1]

The review by Wilson [2] combined arterial and venous leg ulceration from the outset, making a comparison with our review inappropriate. Moreover, Wilson's review merely consecutively summarised the main findings of each study without linking them together in a logical sequence. In contrast, the review by Persoon et al. [1] was strictly subdivided into quantitative and qualitative research methodology. Although Persoon's review basically identified the same major themes as we did, by the use of across-method triangulation – that is, the reconciliation of both types of findings (qualitative and quantitative), the present review provides a more complete picture of this complex phenomenon. The integration of both qualitative and quantitative findings was mainly in the form of embedding, but also in comparing, contrasting, or building on one type of finding with the other. Differences in the number of studies included into the respective review articles might be explained by (1) different in/exclusion criteria as defined by the authors and (2) a different time frame for published papers.

### Critical reflection of the studies

Numerous studies did not undertake a differentiation of participants by ulcer aetiology, yet the aetiology of patient's ulcers has a significant impact on the themes identified. For instance, the quality of pain is known to be associated with aetiology. The stabbing, nocturnal pain relieved by sitting up with legs over the side of the bed is likely to be associated with arterial insufficiency. Otherwise the nagging ache is often associated with venous ulceration. Moreover, the effects on a patient's lifestyle changes may differ depending on the ulcer aetiology. Hence limiting the sample to a single aetiology would have provided further opportunities for building on what is known [33]. Moreover, the vast majority of studies included in the review did not analyse the results according to gender differences. It appeared as if the authors have, as far as possible, suppressed a gender perspective. Failure to consider this issue may weaken the interpretation of research findings [16].

### Implications of the findings

The venous leg ulcer knowledge test employed by Chase et al. [17] revealed knowledge deficits on the part of patients. Nevertheless, educational measures aimed at reducing knowledge deficits cannot be successful without knowledge about the illness experience among caregivers. Hence caregivers need to be sensitised to how the illness is experienced in order to provide adequate health care. The challenge is to move from a focus on wound management to understanding the specific needs of each individual within the context of their life. If these advanced skills are present caregivers can anticipate problems and will be able to provide more sensitive care [26]. Moreover, a vast number of the national guidelines on leg ulcer management do not account for many of the far-ranging effects of leg ulceration identified in this review. Therefore they need to be adjusted in order to improve nursing and medical care in the future.

## Competing interests

The author(s) declare that they have no competing interests.

## Authors' contributions

ORH: developed the search strategy, searched the literature for relevant articles and drafted the whole manuscript. WS: gave advice on the drafted manuscript. MAR: discussed in/exclusion of some articles and gave advice on the drafted manuscript. All authors read and approved the final manuscript.
